# Smart coumarin-tagged imprinted polymers for the rapid detection of tamoxifen

**DOI:** 10.1007/s00216-015-9296-8

**Published:** 2016-02-09

**Authors:** Judith V. Ray, Fosca Mirata, Celine Pérollier, Michel Arotcarena, Sami Bayoudh, Marina Resmini

**Affiliations:** Department of Chemistry, SBCS, Queen Mary University of London, Mile End Road, London, E1 4NS UK; POLYINTELL, Pharma Parc II, Voie de l’Innovation, Chaussée du Vexin, 27100 Val-de-Reuil, France

**Keywords:** Molecularly imprinted polymer, Tamoxifen, 6-Vinylcoumarin-4-carboxylic acid, Clomiphene

## Abstract

A signalling molecularly imprinted polymer was synthesised for easy detection of tamoxifen and its metabolites. 6-Vinylcoumarin-4-carboxylic acid (VCC) was synthesised from 4-bromophenol to give a fluorescent monomer, designed to switch off upon binding of tamoxifen. Clomiphene, a chlorinated analogue, was used as the template for the imprinting, and its ability to quench the coumarin fluorescence when used in a 1:1 ratio was demonstrated. Tamoxifen and 4-hydroxytamoxifen were also shown to quench coumarin fluorescence. Imprinted and non-imprinted polymers were synthesised using VCC, methacrylic acid as a backbone monomer and ethylene glycol dimethacrylate as cross-linker, and were ground and sieved to particle sizes ranging between 45 and 25 μm. Rebinding experiments demonstrate that the imprinted polymer shows very strong affinity for both clomiphene and tamoxifen, while the non-imprinted polymer shows negligible rebinding. The fluorescence of the imprinted polymer is quenched by clomiphene, tamoxifen and 4-hydroxytamoxifen. The switch off in fluorescence of the imprinted polymer under these conditions could also be detected under a UV lamp with the naked eye, making this matrix suitable for applications when coupled with a sample preparation system.

## Introduction

Tamoxifen belongs to a family of drugs known as selective oestrogen receptor modulators (SERM), which are characterised by agonist or antagonist activity depending on the different tissues. Clinically, tamoxifen is widely used for the treatment of breast cancer, as it acts as antagonist by competitively binding to the oestrogen receptors in the breast, preventing further production of tissue and effectively stopping tumours’ growth [1]. However for the past 15 years, tamoxifen has been classified as a prohibited substance by the World Anti-doping Authority (WADA) and the International Olympic Committee (IOC); as a result of its antagonist activity in breast tissues, this drug has been inappropriately used by athletes to mask the side effects of using growth hormones and anabolic androgenic steroids (AAS), well known for causing gynecomastia, i.e. enlargement of breast in man [2]. The exposure of such drug’s abuse requires frequent testing of large numbers of biological samples during sporting events, which is currently not easily achievable.

Detection of tamoxifen and its metabolites is mainly achieved via LC-MS and GC-MS after lengthy hydrolysis, extraction and derivatisation steps [3, 4]. The hydrolysis step is required due to the fact that 95 % of tamoxifen and its metabolites in urine are conjugated to glucuronic acid [5], which makes direct HPLC-UV analysis difficult due to co-elution peaks. 4-Hydroxytamoxifen and 4-hydroxy-3-methoxytamoxifen, two of the main metabolites, do exist in urine [6] but in very low concentrations (10 ng/ml) [7]. Sample preparation with molecularly imprinted polymers (MIP) before HPLC-UV analysis has been demonstrated [7] and allows extraction and enrichment of tamoxifen and tamoxifen polar metabolites with good clean up performance, where the main interferences are removed, compared to other SPE methods. Other methods have also been investigated, and these include flow cytometry [8], video mass spectrometry [9], zero current potentiometry to monitor drug interactions with DNA [10], ultraviolet or fluorescence detection of photocyclisation products [11] and liquid scintillation counting [12]. All these methods require complex instrumentation and highly skilled staff and do not allow screening of large number of samples. There is a clear interest in the development of a detection system that could allow a fast pre-screening of larger number of samples, significantly reducing the costs while also acting as a deterrent.

Molecular imprinting uses a casting procedure to allow the formation of three-dimensional cavities in a polymer matrix, with specific recognition properties. MIPs have been developed in sample preparation prior to HPLC-UV analysis to ensure efficient extraction and enrichment of tamoxifen and its polar metabolites following the hydrolysis step [7] and the stability of the matrix to harsh environments offers an interesting alternative to proteins.

We report here the development of a new MIP for the detection of tamoxifen that contains a smart coumarin-based fluorescent tag that can be quenched upon binding to tamoxifen and 4-hydroxytamoxifen, to be potentially used in association with the SPE system for preparation of the sample. The imprinted polymers show a very good imprinting efficiency, above 20, compared to the non-imprinted matrix, and also the ability to recognise not only the cognate template but also tamoxifen and its metabolite 4-hydroxytamoxifen.

## Experimental

### Materials

4-Bromophenol (97 %), triphenylphosphine (99 %), *tris*(dibenzylideneacetone)dipalladium(0) (97 %), extra dry on molecular sieves tetrahydrofuran (THF) (99.5 %), 2,2′-azobis(2-methylpropionitrile) (99 %) (AIBN), extra dry on molecular sieves dichloromethane (DCM) (99.8 %) and ethyl acetate (99 %) were all purchased from Acros Organic (Geel, Belgium). Dimethyl acetylenedicarboxylate 98 %, triphenyl arsine (98 %) and tributyl(vinyl)tin (96 %) were all purchased from Alfa Aesar (Karlsruhe, Germany). Magnesium sulphate (98 %), ethanol (99.5 %), sodium hydroxide and concentrated hydrochloric acid (37 %) were supplied by Carlo Erba (Val de Reuil, France.). Sodium fluoride (99 %), sodium chloride (98 %), methacrylic acid (MMA) (99 %), clomiphene citrate (analytical standard 42 % *cis* and 58 % *trans*), 4-hydroxy tamoxifen (>70 %) and EGDMA (98 %) were purchased from Sigma Aldrich (France and UK). Tamoxifen citrate (99 %) was purchased from LKT lab Inc. Acetonitrile (99.9 %) (ACN) was obtained from Fischer Chemicals; acetic acid (99.7 %) and methanol (99.9 %) were all HPLC grade and supplied by Carlo Erba (Val de Reuil, France). AIBN was recrystallised from methanol before use. Clomiphene and tamoxifen were isolated from their corresponding citrate salts using sodium bicarbonate (10 mM) and extracted with DCM. Falcon 96-microplates were purchased from SLS.

### Synthesis of methyl 6-bromocoumarin-4-carboxylate

4-Bromo-phenol (18.26 g, 0.11 mol) and triphenylphosphine (27.66 g, 0.11 mol) were dissolved in dry DCM (100 ml) and cooled in a salt/ice bath to −5 °C. Dimethyl acetylenedicarboxylate (15 g, 0.11 mol) was added to the mixture drop-wise under nitrogen. On addition, the solution immediately turned red. The mixture was stirred at −5 °C for 1 h before being refluxed at 40 °C for approximately 100 h. The solvent was evaporated and the red/brown residue was recrystallised from ethanol. The orange precipitate was filtered off under suction and rinsed with ethanol to give a creamy/pale yellow solid. The product was purified on silica via automated flash chromatography (95:5 DCM, petroleum ether) giving the desired product with a 25 % yield. The 6-bromocoumarin 4-carboxylate was confirmed by HPLC-MS *m*/*z* (M + H)^++^: 285 at 18.2 min.

### Synthesis of methyl 6-vinylcoumarin-4-carboxylate

6-Bromocoumarin 4-carboxylate (6.82 g, 24 mmol) and triphenyl arsine (1.48 g, 4.8 mmol) were dissolved in dry THF (100 ml) and the palladium catalyst (550 mg, 0.6 mmol) was added to the mixture under nitrogen. The mixture was stirred for 10 min, before adding tributyl(vinyl)tin (8.8 ml, 9.54 g, 30 mmol) and refluxing for 125 h at 70 °C. The reaction solution was left to cool to room temperature before adding 35 ml saturated sodium fluoride solution and 17 ml distilled water and stirring for 1 h. Following filtration, the solution was extracted with DCM (2 × 50 ml) and washed with saturated salt solution (2 × 50 ml). The extracts were dried over magnesium sulphate, filtered and evaporated under suction. The solid crude product was purified on silica via automated flash chromatography using an eluent of 5:95 ethyl acetate/DCM to give the desired product as a yellow solid (49 % yield). Purity of methyl 6-vinylcoumarin-4-carboxylate was confirmed via HPLC-MS: *m*/*z* (M + H)^+++^: 233 at 17.9 min.

### Synthesis of 6-vinylcoumarin-4-carboxylic acid (VCC)

Methyl 6-vinylcoumarin-4-carboxylate (3.1 g, 13 mmol) was dissolved in a solution of 42 ml THF and 108 ml ethanol. Sodium hydroxide (25 ml, 2 M) was added to the reaction and the solution was heated (to 40 °C) under nitrogen for 24 h. Solvents were removed under vacuum and 60 ml of water were added to the residue, which was washed with DCM (2 × 100 ml) and ethyl acetate (2 × 100 ml), before being acidified with concentrated hydrochloric acid. The aqueous phase was then extracted with ethyl acetate (3 × 50 ml) and the combined organise phase washed with a saturated NaCl solution (2 × 50 ml). The organic phase was dried over magnesium sulphate and evaporated to dryness to give 6-vinylcoumarin-4-carboxylic acid (96 % yield). HPLC-MS *m*/*z* (M + H)^+++^: 219 at 13.9 min. ^**1**^**H**-**NMR** δ(ppm) (400 MHz, DMSO): 8.18 (1H, d, J = 2), 7.84 (1H, dd, J = 8, 2) 7.44 (1H, d, J = 8), 6.85 (1H, s), 6.84 (1H, dd, J = 18, 11), 5.86 (1H, d, J = 18), 5.35 (1H, d, J = 11) ^**13**^**C**-**NMR** δ( ppm) (400 MHz, DMSO): 165.3(1C), 159.5 (1C), 153.3 (1C), 143.9 (1C), 135.3 (1C), 133.6 (1C), 129.5 (1C), 124.5 (1C), 118.1 (1C), 117.2 (1C), 115.8 (1C) 115.2 (1C).

### Synthesis of polymers

6-Vinylcoumarin-4-carboxylic acid (1.1 mmol), MMA (3.34 mmol), ethylene glycol dimethacrylate (EDGMA; 22.2 mmol) and clomiphene (1.1 mmol, for the MIP) were dissolved in dry acetonitrile to give a clear solution (>10 ml). Nitrogen was bubbled through the mixture for 10 min, followed by the addition of AIBN (0.20 mmol). The sealed flask was placed in an oil bath at 50 °C for 2 days. The solid material was ground into large chunks and was extracted five times using an eluent of 92.5 % methanol, 2.5 % water, 5 % acetic acid and then washed two further times with 100 % methanol. The material was subsequently ground and sieved between 80 and 45 μm before being extracted and washed again with the same eluent.

### Characterization of the imprinting factor by chromatography

The MIP and non-imprinted polymer (NIP) bulk polymers were further ground and sieved to between 45 and 25 μm. Approximately 300 mg of polymer was sonicated in 16 ml chloroform and 4 ml of methanol before being packed into a column (GraceSmart RP18 3u (150 × 2.1 mm)) with methanol. The columns were pre-conditioned with ACN for 1 h before injecting 1 and 10 mM solutions of clomiphene and tamoxifen into each column. The columns were eluted with a 5.5 % acidic aqueous solution (comprising 580 μl acetic acid in 500 ml water, brought to pH 4 with sodium hydroxide) in ACN. Flow rate was 1 ml/min, column UV detection at 254 nm, operating at 21 °C. Each injection was repeated in triplicate. The IF was calculated using the following equations, where *t* is the retention time of the analyte, *t*_0_ is the retention time of the void marker and *k* is the retention factor.

$$ \begin{array}{ll}k=\frac{t-{t}_0}{t_0}\hfill & \mathrm{IF}=\frac{k_{\mathrm{MIP}}}{k_{\mathrm{NIP}}}\hfill \end{array} $$

### Fluorescence quenching of VCC with analytes

The fluorescence measurements were carried out on a FluoroMax-3 fluorimeter. Excitation wavelength was 345 nm, emission at 521 nm with emission slit 1.5. A 5.93 × 10^−4^ M stock solution of VCC, a 1.48 × 10^−2^ M stock solution of tamoxifen and clomiphene and a 5.16 × 10^−3^ M stock solution of 4-hydroxytamoxifen were prepared in acetonitrile.

To 1 ml of VCC stock solution, the appropriate volume of analyte solution (to give 0.5, 0.75 and 1 eq) was added followed by addition of ACN to give a total volume of 2 ml. The solutions were kept at room temperature for 5 min and the fluorescence was then measured. Each experiment was repeated in triplicates and the average of the three datasets was used, presented together with the standard deviation.

### Fluorescence quenching of MIP with analytes

The fluorescence measurements were carried out on a FLUOstar OPTIMA plate reader. The number of intervals was set at 150 with 10 flashes per well and 2.20 s as interval time. The excitation filter used was 340-10 and the emission filter was 510-20 with a gain of 2000. A 5.9 × 10^−3^ M stock solution of tamoxifen and clomiphene and a 5.16 × 10^−3^ M stock solution of 4-hydroxytamoxifen were prepared in acetonitrile. To 1 mg of MIP, weighted directly into a well, 200 μl of acetonitrile and the appropriate volume of analyte solution (to give 0.25, 0.5, 0.75 and 1 eq) were added. The solutions were kept at room temperature for 5 min and the fluorescence was then measured. Each experiment was repeated in duplicates and the average of the two data was used, presented together with the standard deviation.

### Instruments and analytical conditions

A Finnigan surveyor HPLC-MS, using hypersil gold reverse phase (50 × 2.1 mm) column was used for all mass spectrometer measurements of the organic molecules synthesised. The column was pre-conditioned with 100 % (0.1 %) formic acid solution. The 0.1 % formic acid solution was pumped through the column on a gradient with ACN at a rate of 0.2 ml per minute.

The Dionex ASE 350 accelerated solvent extraction machine was used to extract/wash all bulk polymers formed. A FluoroMx-3 fluorimeter and a FLUOstar OPTIMA plate reader were used to perform VCC and MIP fluorescence measurements, respectively. The 1666 slurry packer from Alltech was used to load the HPLC columns with the MIPs and NIPs that had been prepared for analysis.

A HPLV-UV Thermofinnigan Spectra System was used for the determination of the imprinting factor. The retention time of the clomiphene in the presence of MIP/NIP was obtained with a mobile phase of 5:95 acetic acid/acetonitrile solution.

## Results and discussion

For the purpose of synthesising a molecular imprinted polymer for the detection of tamoxifen (1), the chlorinated analogue clomiphene (2) was chosen as template, as it had been previously shown by our group to be a good mimic of the drug. When developing imprinted polymers for analytical purposes, an analogue of the analyte is often used to imprint the polymer, as this prevents any traces of the drug being found, as a result of some template remaining enclosed in the polymer matrix and bleeding during analysis [7].

**Fig. 1:**
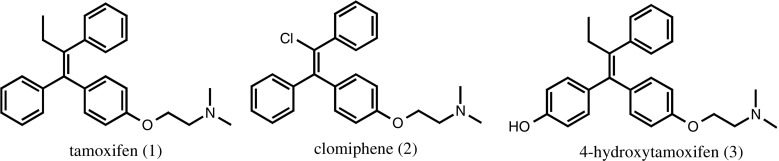
Structure of the target analytes—tamoxifen (*1*), the template—clomiphene (*2*) and the metabolite—4-hydroxytamoxifen (*3*)

Clomiphene (2) is structurally very similar to the target analyte tamoxifen (1), with three phenyl groups providing multiple opportunities for hydrophobic interactions, and the replacement of the ethyl group with the chlorine atom being the only structural difference. This is not expected to have a significant impact on molecular recognition, as the main interactions between template and monomer will derive from the ionic bond of the tertiary amine in tamoxifen and the carboxylic acid in the polymer, together with the hydrophobic interactions derived from π-π stacking. Additionally, studies were also carried out using 4-hydroxytamoxifen (3), one of the main metabolites of tamoxifen (Fig. 1).

In order to develop an imprinted polymer that can be used in an optical sensor for the detection of tamoxifen, a monomer with specific optical properties that change upon drug binding was required. The interaction between the template and the functional monomer in an imprinted polymer plays a key role in determining the specificity and rebinding characteristics of the cavities. These interactions can take the form of covalent, semi-covalent or non-covalent bonds such as dipole-dipole interactions, hydrogen bonding, electrostatic interactions and Van der Waals forces. Although in general the stronger the interactions are, the higher the imprinting efficiency is, successful imprinted polymers have been obtained relying only on low energy interactions like hydrogen bonds [13].

6-Vinylcoumarin-4-carboxylic acid was selected as functional monomer for this work, as it satisfied two important requirements: (i) the presence of the COOH group, which allows strong ionic interactions to take place with the tertiary amine unit of tamoxifen, 4-hydroxytamoxifen and clomiphene; (ii) the strong fluorescence of the coumarin unit, which was previously shown to be easily quenched when in contact with ephedrine [14].

### Studies of the interaction between 6-vinylcoumarin-4-carboxylic acid and clomiphene, tamoxifen and 4-hydroxytamoxifen

**Fig. 2:**
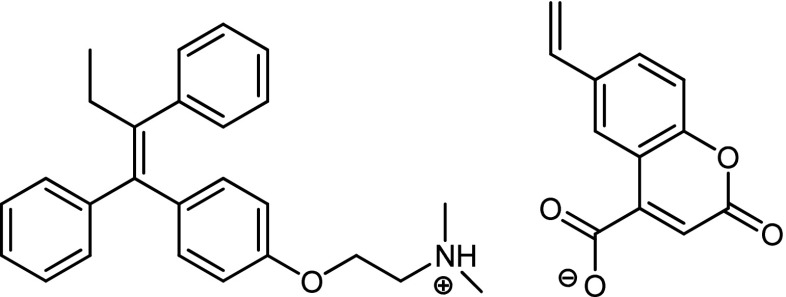
Ionic interaction between tamoxifen and 6-vinylcoumarin-4-carboxylate

Methacrylic acid (MAA) has been previously shown to have a broad applicability as a functional monomer in MIPs due to its ability to act as proton donor as well as a hydrogen bond donor and acceptor. Electrostatic interactions and hydrogen bonds play a key role in the binding of the template. Previous work involving methacrylic acid and tamoxifen demonstrated that the ionic interaction between the acid group in MAA and the tertiary amine in tamoxifen played a key role in determining molecular recognition and rebinding [7]. By analogy, the same interaction was expected with 6-vinylcoumarin-4-carboxylic acid which contains a carboxylic group and whose fluorescent characteristics change when involved in non-covalent bonding (Fig. 2).

**Fig. 3:**
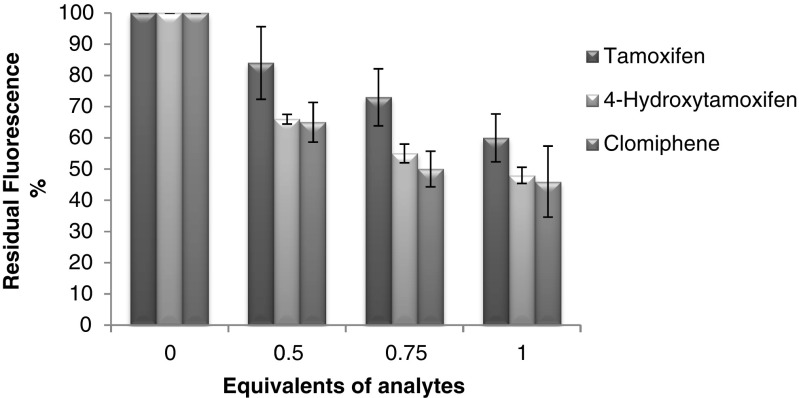
Fluorescence quenching of 6-vinylcoumarin-4-carboxylic acid monomer (VCC), following the addition of the analytes tamoxifen, 4-hydroxytamoxifen and clomiphene in different quantities ranging from 0 to 1 equivalent in ACN. Fluorescence spectra recorded at 521 nm following excitation at 345 nm. [VCC] = 2.97 × 10^−4^ M, *V*_tot_ = 2 ml, incubation time 5 min, experiments repeated in triplicates

The fluorescence quenching of the functional monomer VCC as a result of its interactions with clomiphene, tamoxifen and also 4-hydroxytamoxifen was studied. VCC has a strong fluorescence emission at 521 nm when excited at 345 nm. Solutions of VCC (5.9 × 10^−3^ M) were mixed with varying volumes of analytes stocks in acetonitrile, to give a final monomer to analytes ratio ranging from 1:0 to 1:1. All the samples were allowed to mix for 5 min at room temperature before being analysed. The combined data for all three analytes are shown in Fig. 3 and are presented as percent of residual fluorescence following addition of the quencher solution. As the equivalents of analytes are increased from 0 to 0.5, 0.75 and 1, there is a progressive decrease in residual fluorescence. All three analytes are able to quench the VCC fluorescence, with 1 equivalent resulting in approximately 50 % quenching. It is interesting to observe that the chemical structure of the analytes clearly has some effect on the interaction and the extent of the quenching, with clomiphene appearing to be the best quencher (54 % quenched) compared for instance to tamoxifen (40 % quenched), when all the other experimental conditions are kept constant. This is an important factor to be considered when the polymers are evaluated.

### Polymer synthesis and binding interaction studies with clomiphene and tamoxifen

Detection of tamoxifen using a sensor requires recognition and binding of the analyte to take place in an aqueous environment, therefore the polymeric matrix needs to be as hydrophilic as possible, to facilitate the process. Methacrylic acid was added to the polymer preparation as the backbone monomer. The presence of the carboxylic groups was expected to contribute to the binding, as previously demonstrated [7] without any detrimental effect on the fluorescence properties of the functional monomer.

The chemical structure of the cross-linker and its concentration play a very important role in determining the binding properties of the imprinted polymers. The higher the percentage of cross-linker, the more rigid the polymer structure and also the cavities are, and the more likely they are to retain their three-dimensional shape. EDGMA is the molecule of choice in many imprinted polymers and it was chosen as the cross-linking agent as it had proved to be well incorporated when imprinting with clomiphene in previous formulations [7]. The polymerizations were carried out in acetonitrile, the same solvent that was used for the interaction studies between VCC and clomiphene, using AIBN as the initiator, polymerising for 2 days at 50 °C.

A number of different polymers were prepared using a variety of ratio of monomers to crosslinker, with the aim of maximising the incorporation of the VCC functional monomer while retaining a good degree of cross-linking and solubility. Initial formulations were prepared without any methacrylic acid; however, the overall solubility of the polymer was not sufficient for the desired applications. Following a number of attempts, the best imprinted preparation (MIP) was obtained by using the ratio of 1:1:3:20, T/FM/BM/XL where T = clomiphene, FM = 6-vinylcoumarin-4-carboxylic acid, BM = MAA and XL = EDGMA, with 80 % cross-linker and a 1:1 ratio between clomiphene and VCC. The final ratio of monomers to porogenic solvent, ACN, was 5:6 (*w*/*v*). The corresponding NIP was prepared under identical conditions but without the presence of the clomiphene template.

At the end of the polymerization reaction, the bulk polymers were broken into large pieces that were washed with a solution of 2.5 % acetic acid in methanol, to remove the template from the MIP. HPLC analysis of the extraction solutions showed that at this stage 34 % of the template had been removed, suggesting that a large portion of the template was still inside the matrix. Both set of polymers were subsequently ground and sieved, with particles between 80 and 45 μm, before being extracted and washed again with the same eluent, to ensure the complete removal of all templates. Further grinding was carried out to obtain particles between 45 and 25 μm, and final HPLC analysis of the extraction solution indicated that 95 % of the template was removed. The incorporation of VCC in the polymers was estimated by fluorescence using a reference line. In order to evaluate the different binding capacities of the polymers towards the analytes, both MIP and NIP were pressure-loaded each into a chromatography column. A dilute solution of acetone in ACN was injected into the column as a void volume marker. Injections of 10 mM solutions of analyte in ACN allowed binding to take place. Monitoring of the elution profile in 100 % ACN showed that no template was being released even after 1 h. When a solution of 5 % acetic acid in ACN was used, the analytes were successfully eluted as a result of the disruption to the binding interactions by the acetic acid. The same experiment was carried out with clomiphene and tamoxifen on both MIP and NIP columns. Each injection was repeated in triplicate with SD <5 %. The imprinting factors (IF) for both template and target analyte obtained (Table 1) showed the superior rebinding capability of the MIP.

**Table 1:** Sample volume 20 μl, flow rate 1 ml/min, column dimension = 150 mm × 2.1 mm. Detection at 254 nm, 21 °C. Void volume marker was acetone, the retention factor and imprinting factor (IF) were calculated as described in the experimental

Analyte	Concentration in ACN (mM)	*k*_NIP_	*k*_MIP_	IF
Clomiphene	10	2.9	63.0	21.7
Tamoxifen	10	2.3	43.0	18.7

The data shown in Table 1 indicate that the imprinted polymer has a superior molecular recognition capacity towards clomiphene compared to the non-imprinted polymer, with an IF = 21.7. The same polymer shows excellent selectivity also towards tamoxifen, with an imprinting factor of 18.7. Comparison of MIP rebinding data for clomiphene and tamoxifen demonstrated that the affinity of the MIP for clomiphene, the cognate template, is 32 % higher than for tamoxifen, as a result of the imprinting process, while the NIP has very similar values for both analytes, showing very little rebinding affinity, with values of *k*_NIP_ equal to 2.9 and 2.3, respectively. The data demonstrate that the imprinting process successfully generated a polymer matrix with very good molecular recognition properties for both clomiphene and tamoxifen, while the non-imprinted polymer, as expected, does not appear to rebind significantly any of the analytes. The next step focused on the characterisation of the fluorescent properties of the imprinted polymer.

### Fluorescence quenching studies with imprinted polymer

**Fig. 4:**
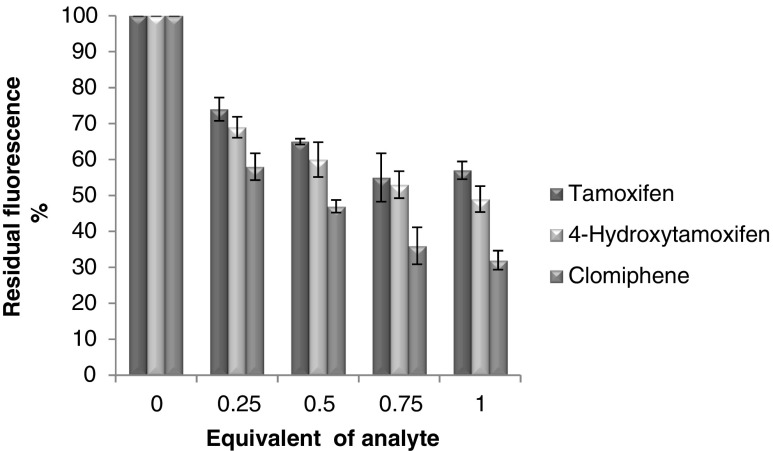
Residual fluorescence of MIP (5 mg/ml in ACN, 1.18 × 10^−3^ M in VCC), following the addition of varying equivalents of analytes (tamoxifen, 4-hydroxytamoxifen and clomiphene). Fluorescence spectra recorded at 521 nm following excitation at 345 nm. Data are presented as percentage of residual fluorescence compared to the control

In order to study how the fluorescence properties of the VCC were influenced as a result of being incorporated into the imprinted matrix, a series of experiments were carried out using MIP together with tamoxifen, 4-hydroxytamoxifen and clomiphene. Solutions of the imprinted polymer (5 mg/ml in ACN) were placed in 96-well plate and various amounts of analytes were added to give a VCC-analyte ratio ranging from 0 to 0.25,0.5, 0.75 and 1. The solutions were left for 5 min after which the residual fluorescence was measured using a plate reader. The data presented in Fig. 4 for all three analytes show that the interaction of the polymer with increasing amounts of analytes results in all cases in progressive quenching of its fluorescence. Interestingly in all cases, the difference in percentage of fluorescence quenched is consistent with the data previously obtained with VCC alone, with clomiphene being the strongest quencher, followed by 4-hydroxytamoxifen and then tamoxifen.

### Visual detection of clomiphene with MIP compared to NIP

**Fig. 5:**
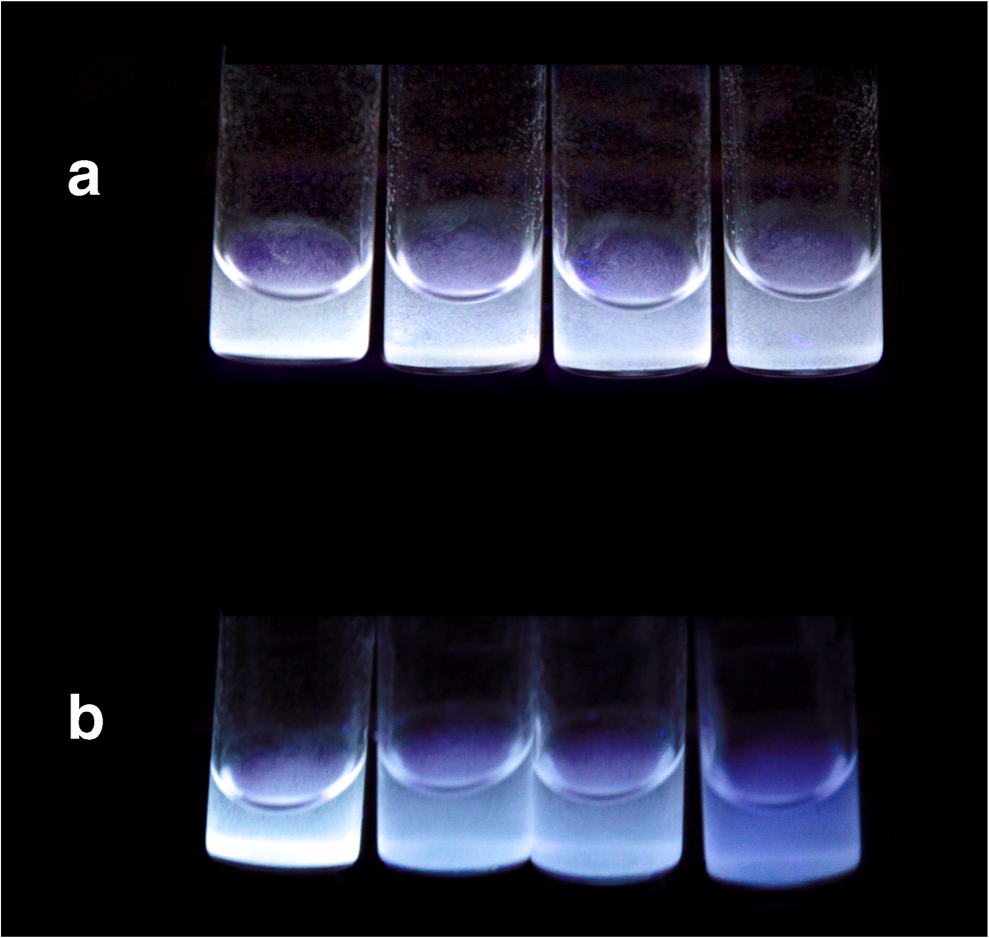
Visual detection of clomiphene with NIP (**A**) and MIP (**B**) by using a standard 365 nm UV light (2 mg of polymer in 500 μl of ACN). From *left to right*, ratio of fluorophore:clomiphene 1:0, 1:0.5, 1:0.75, 1:1. **A** shows no significant variation in fluorescence by increasing the amount of clomiphene, while **B** shows a clear quenching of fluorescence passing from 0 to 1 equivalents of clomiphene

In order to evaluate the feasibility of using the imprinted polymers as materials for the development of a visual sensor, a quenching experiment with clomiphene was carried out with both MIP and NIP using a standard 365 nm UV light. For each polymer, four glass vials were prepared containing a suspension of 2 mg of either MIP or NIP in 400 μl of ACN (5 mg/ml of polymer, 1.18 × 10^−3^ M in VCC). To each vial, a combination of ACN and clomiphene stock solution (1.42 × 10^−2^ M) was added to give a total volume of 500 μl and a ratio of VCC to clomiphene ranging from 1:0 to 1:1. The mixtures were allowed to stand for 5 min at room temperature and were then placed under the UV lamp. Figure 5 shows the fluorescence of the vials. It can be clearly seen that while in the case of MIP (Fig. 5B) there is evidence of quenching, increasing from 0 to 1 equivalents of clomiphene, in the case of NIP the fluorescence remains unaltered (Fig. 5A).

## Conclusions

In this work, VCC and clomiphene were successfully used with molecular imprinting to create a ‘smart’ switchable polymer, with fluorescent binding sites able to recognise not only the cognate substrate clomiphene but also tamoxifen and 4-hydroxytamoxifen. The imprinted polymer was shown to have excellent molecular recognition properties in ACN towards clomiphene and tamoxifen, with imprinting efficiencies of 21.7 and 18.7, respectively, while the NIP did not show any significant binding affinity, as determined by HPLC. The study of fluorescence quenching of the MIP by clomiphene, tamoxifen and 4-hydroxytamoxifen show progressive quenching as a function of increasing analyte concentration in ACN. This is consistent with data obtained with VCC and the analytes alone. Finally, the MIP was used for a visual detection test in comparison with the NIP. The results show that the quenching of the MIP can be seen with naked eye, while the NIP fluorescence remains unaltered. This represents a significant first step in creating a visual sensor for drugs, allowing their presence and potential misuse to be detected and assessed by a lay person. The fact that tamoxifen and its metabolites are essentially insoluble in water and are found in urine as conjugates of glucuronic acid means that the hydrolysis step followed by concentration using SPE, as previous reported, is a necessary step. The polymer obtained in this work is expected to work coupled to the SPE cartridge, therefore allowing the detection of the analytes to occur in acetonitrile, where the binding is maximised. In order to increase the sensitivity of the polymer, the next step will require the preparation of nano-sized polymeric particles. By increasing the surface-to-volume ratio and hence exposing more active sites, a greater quantity of the smart coumarin tag will be available for the drug to rebind, therefore providing a lower detection limit.
